# A retrospective multicenter cohort study of the association between anti-Factor Xa values and death, thromboembolism, and bleeding in patients with critical COVID-19

**DOI:** 10.1186/s12959-023-00541-z

**Published:** 2023-10-02

**Authors:** Sandra Jonmarker, Jacob Litorell, Felix Alarcon, Kais Al-Abani, Sofia Björkman, Maria Farm, Jonathan Grip, Mårten Söderberg, Jacob Hollenberg, Rebecka Rubenson Wahlin, Thomas Kander, Liivi Rimling, Johan Mårtensson, Eva Joelsson-Alm, Martin Dahlberg, Maria Cronhjort

**Affiliations:** 1 Department of Clinical Science and Education, Karolinska Institutet, Södersjukhuset, Stockholm, Sweden; 2 Department of Anaesthesia and Intensive Care, Södersjukhuset, Stockholm, Sweden; 3 Department of Emergency and Reparative Medicine, Karolinska University Hospital, Stockholm, Sweden; 4 Department of Clinical Science, Anaesthesiology and Intensive Care, Lund University, Skåne University Hospital, Lund, Sweden; 5 Department of Molecular Medicine and Surgery, Karolinska Institutet, Stockholm, Sweden; 6 Department of Clinical Chemistry, Karolinska University Hospital, Stockholm, Sweden; 7 Department of Perioperative Medicine and Intensive Care, Karolinska University Hospital, Stockholm, Sweden; 8 Department of Clinical Science, Intervention and Technology (CLINTEC), Karolinska Institutet, Stockholm, Sweden; 9 Department of Internal Medicine, Södersjukhuset, Stockholm, Sweden; 10 Department of Clinical Science and Education, Centre for Resuscitation Science, Karolinska Institutet, Södersjukhuset, Stockholm, Sweden; 11 Department of Physiology and Pharmacology, Section of Anaesthesia and Intensive Care, Karolinska Institutet, Stockholm, Sweden; 12 Department of Surgery, Södersjukhuset, Stockholm, Sweden

**Keywords:** COVID-19, Thromboembolism, Hemorrhage, Heparin, low-molecular-weight, Critical care

## Abstract

**Background:**

Patients with critical COVID-19 have a high risk of thromboembolism, but intensified thromboprophylaxis has not been proven beneficial. The activity of low-molecular-weight heparins can be monitored by measuring anti-Factor Xa. We aimed to study the association between anti-Factor Xa values and death, thromboembolism, and bleeding in patients with critical COVID-19.

**Method:**

This retrospective cohort study included adult patients with critical COVID-19 admitted to an intensive care unit at three Swedish hospitals between March 2020 and May 2021 with at least one valid peak and/or trough anti-Factor Xa value. Within the peak and trough categories, patients’ minimum, median, and maximum values were determined. Logistic regressions with splines were used to assess associations.

**Results:**

In total, 408 patients had at least one valid peak and/or trough anti-Factor Xa measurement, resulting in 153 patients with peak values and 300 patients with trough values. Lower peak values were associated with thromboembolism for patients’ minimum (*p* = 0.01), median (*p* = 0.005) and maximum *(p* = 0.001) values. No association was seen between peak values and death or bleeding. Higher trough values were associated with death for median (*p* = 0.03) and maximum (*p* = 0.002) values and with both bleeding (*p* = 0.01) and major bleeding (*p* = 0.02) for maximum values, but there were no associations with thromboembolism.

**Conclusions:**

Measuring anti-Factor Xa activity may be relevant for administrating low-molecular-weight heparin to patients with critical COVID-19. Lower peak values were associated with an increased risk of thromboembolism, and higher trough values were associated with an increased risk of death and bleeding. Prospective studies are needed to confirm the results.

**Trial registration:**

The study was retrospectively registered at Clinicaltrials.gov, NCT05256524, February 24, 2022.

**Supplementary Information:**

The online version contains supplementary material available at 10.1186/s12959-023-00541-z.

## Background

Patients with critical COVID-19 have a higher risk of arterial and venous thromboembolism (TE) than other critically ill patients, with a reported incidence of 16 – 69% in several observational studies [1–6]. The optimal thromboprophylactic dose differs with severity and timing within the disease course. Prophylactic anticoagulation with high doses has been shown to decrease mortality in patients with moderately severe COVID-19 [7–9]. However, no dosing regimen has shown superior survival rates for patients treated in the intensive care unit (ICU) [10–12]. High-dose anticoagulation may cause more bleeding than the standard prophylactic dose, therefore, guidelines recommend against intensified thromboprophylaxis for patients with critical COVID-19 [13].

Low-molecular weight heparin (LMWH) is the preferred drug for preventing thromboembolic complications in the ICU [14]. The activity of LMWH can be monitored by quantifying anti-Factor Xa (aFXa) in plasma [15]. This can be measured as a peak value by taking the blood sample when the concentration of LMWH is expected to be highest or as a trough value just before the next dose. Target values for the treatment of venous TE are agreed upon, but neither peak nor trough target values for thromboprophylaxis are clear [16]. For the subgroup of critically ill patients, there is an even greater uncertainty regarding optimal prophylactic aFXa values, and studies have shown trough values to be consistently low [17].

Not only are target values not established, but aFXa has also been questioned as a surrogate marker for the effect of LMWH, as many studies have failed to determine an association between aFXa and the risk for TE and bleeding [18–21].

In summary, patients with critical COVID-19 have a high risk of TE, which is associated with increased mortality [3]. Uniformly increasing LMWH from standard low thromboprophylaxis to intermediate and high doses has not been proven beneficial. During the pandemic, the perceived abnormal coagulation in COVID-19 patients resulted in a large number of aFXa analyses in our hospitals. This was done even though evidence is lacking on how to interpret results and how to potentially adjust the dose of LMWH. Whether aFXa-guided thromboprophylaxis can improve outcomes for patients with critical COVID-19 is yet unknown. The aim of our study was to investigate the association between aFXa values and death, TE, and bleeding in patients with critical COVID-19 treated with thromboprophylactic LMWH.

## Method

### Study overview and patients

All adult patients with critical COVID-19 (verified by a polymerase chain reaction test) admitted to an ICU at three Swedish hospitals (Södersjukhuset and Karolinska University Hospital in Stockholm and Skåne University Hospital in Lund) between March 1, 2020, and May 30, 2021, treated with thromboprophylactic LMWH, were eligible. Patients were included on their first admission to the ICU. If a patient was transferred directly from one ICU to another, this was considered the same admission.

Patients were excluded if they had the outcomes of TE (with ongoing treatment) or major bleeding at ICU admission, as LMWH dosing could not be assumed to be prophylactic. Patients initially admitted to an ICU at other hospitals, where we did not have access to the electronic health record (EHR) or Patient Data Management System (PDMS), were excluded.

Patient data were manually and automatically extracted from the EHRs (Take Care®, CompuGroup Medical, Koblenz, Germany and Melior®, Cerner Corporation, Missouri, United States) and PDMSs (Clinisoft®, GE Healthcare, Illinois, United States and IntelliSpace Critical Care and Anaesthesia, Philips Medizin Systeme, Böblingen, Germany). The manual review was performed by five investigators guided by instructions and definitions of patient characteristics, chronic medication, comorbidities (international classification of diseases, 10^th^ revision), treatment provided in the ICU and outcomes. If interpretation of the EHR was difficult, the investigator was instructed to discuss with a more experienced clinician involved in the study. With the use of programming software (QlikView®, 1993–2023, QlikTech International AB, Pennsylvania, United States), data on aFXa values were generated based on drug records with time stamps for administration and laboratory data with time stamps for blood sampling.

The study was approved by the Swedish Ethical Review Authority, registration number 2020–01302 with amendment 2020–02890 for patients included at Södersjukhuset and Karolinska University Hospital, and registration number 2014/916, amendment 2018/866 and 2020–06674 for patients included at Skåne University Hospital. The need for informed consent was waived. Approval for collecting patient data was given by the hospital jurisconsult, and record keeping was performed in accordance with the General Data Protection Regulation. The study was retrospectively registered at Clinicaltrials.gov, NCT05256524, February 24, 2022.

### LMWH, type and dose

Patients were treated with doses and types of LMWH according to local guidelines (Supplement, Local treatment guidelines). For the majority of the study period, a twice daily regime was standard. Patients were grouped according to their initial dose when admitted to the ICU. Regimes of anticoagulation of LMWH were categorized as follows; low dose of LMWH: 2500-4500 international units (IU) daily for tinzaparin, 2500-5000 IU daily for dalteparin or ≤ 40 mg daily for enoxaparin; intermediate dose of LMWH: > 4500 IU but < 175 IU/kg of body weight daily for tinzaparin, > 5000 IU but < 200 IU/kg of body weight daily for dalteparin, or > 40 mg but < 2 mg/kg of body weight daily for enoxaparin; and high dose of LMWH: ≥ 175 IU/kg of body weight daily for tinzaparin, ≥ 200 IU/kg of body weight daily for dalteparin, or ≥ 2 mg/kg of body weight daily for enoxaparin. At Södersjukhuset, tinzaparin was used, at Karolinska University Hospital, dalteparin was used, and at Skåne University Hospital, enoxaparin was used. Patients who were transferred between hospitals in Stockholm may have been treated with both tinzaparin and dalteparin during their ICU stay. In this case, patients were categorized to the LMWH used when they had their valid aFXa values.

### aFXa

aFXa was analysed using standard local routines. Blood was sampled from venous punctures or arterial lines and collected in citrated tubes (3.2%, 0.109 mol/L). Samples were centrifuged within one hour. Platelet-free plasma was obtained by a two-step centrifugation protocol at 3000 × g for ten minutes in the Stockholm laboratories and by a single-step centrifugation at 2000 × g for 20 min in the Skåne laboratory. Analysis of aFXa was performed using the BiophenTM Heparin LRT reagent (Hyphen BioMed, Neuville-sur-Oise, France) on the Sysmex CS-5100 automated coagulation analyser (Sysmex Corporation, Kobe, Japan).

The first aFXa to be analysed for each patient was sampled after at least four doses of LMWH when a steady state was assumed. aFXa was sampled as both trough and peak values. At Södersjukhuset and Karolinska University Hospital, trough values were most commonly used, while at Skåne University Hospital, peak values were almost exclusively used. Peak values were defined as blood sampled at 3 (± 1) hours after the administration of subcutaneous LMWH, and trough values were defined as blood sampled at 12 (± 2) hours after the subcutaneous administration of LMWH according to the two-dose regimen. Values after a diagnosis of TE or major bleeding and after 28 days were excluded.

The Stockholm laboratories measured a range between 0.05 and 1.8 kIU/L; any values below 0.05 were set to 0.049, and values above 1.8 were set to 1.801. In the Skåne laboratory, the measuring range was between 0.1 and 2.4 kIU/L; values below 0.1 were set to 0.099, and values above 2.4 were set to 2.401.

Within the categories peak and trough, samples of aFXa were used to generate minimum, median and maximum values [21]. The minimum and maximum values could be values from any time during the ICU stay, however, the median was calculated using only values from the first 14 days. If a patient only had one value, this was classified as both minimum and maximum and as median if sampled during the first 14 ICU days. With this categorization, all patients had between two (minimum and maximum for either peak or trough) and six values (minimum, median and maximum for both peak and trough) to be analysed. The minimum and maximum values at any time during the ICU stay were chosen to decrease the risk of a lag between the value of aFXa and diagnosis hindering the identification of an association. The median value during the first 14 days was chosen because we hypothesized that LMWH activity early on during the ICU stay may be most important for the association with mortality.

### Outcomes

The primary outcome was death within 90 days, and secondary outcomes were TE, bleeding and major bleeding within 28 days. Pulmonary embolism/thrombosis (PE/PT) and DVT were considered venous TE, and ischemic stroke was considered arterial TE. PE/PT and ischemic stroke were defined as a diagnosis verified by computed tomography, and DVT was defined as verified by ultrasound or computed tomography. The reason for broadening the outcome of PE to PE/PT is reports of occlusions of the pulmonary arteries without findings of thrombus in the veins of the lower extremities in COVID-19 patients. Since lower extremities are usually where the embolizing thrombus is formed, it has been speculated that COVID-19 patients, in addition to PE, also suffer in situ thrombosis in the lungs [22]. All investigations for TE were performed at the discretion of the treating clinician.

Bleeding events were categorized according to the World Health Organization bleeding scale, and grade 3 and 4 bleedings were considered major bleedings [23–25].

### Statistical analysis

Continuous variables are presented as medians with interquartile ranges (IQRs). Categorical variables are expressed as numbers and proportions (percentage). Logistic regression was used to analyse aFXa as a continuous variable. aFXa was modelled as a spline function with three knots, as we made no assumption about the nature of the relationship between aFXa and the outcomes (linear vs. nonlinear). The Wald test was used to assess whether the change in risk of outcome was significantly associated with aFXa values in the spline model, with the null hypothesis that all of the spline coefficients were null.

Chi-square was used when analysing different cut-offs of aFXa and presented as odds ratios (OR) with corresponding 95% confidence intervals (CI).

When assessing for confounders, only renal function was identified to be potentially associated with both aFXa and the outcomes. Renal function was therefore added to the model. An estimated glomerular filtration rate (eGFR) based on sex, age and baseline creatinine was calculated according to the Chronic Kidney Disease Epidemiology Collaboration (CKD-Epi) equation [26]. The distribution of normality was tested using the Shapiro–Wilk test. The correlation between aFXa and eGFR was assessed with Spearman's correlation test. Three regression lines were added to visualize the risk for patients with eGFR 60, 90, and 120 ml/min/1.73m^2^. The Kruskal–Wallis test was used to compare differences between groups for continues variables and Dunn’s test for pairwise multiple comparisons. No method was used to adjust for multiple testing except when using Dunn’s test and adjustment was done by the Bonferroni method.

A two-sided *p* value < 0.05 was considered statistically significant. Analyses were performed in R v 4.2.2 (R Core, 2017. R: A language and environment for statistical computing. R Foundation for Statistical Computing, Vienna, Austria).

## Result

### Patients

**Fig. 1:**
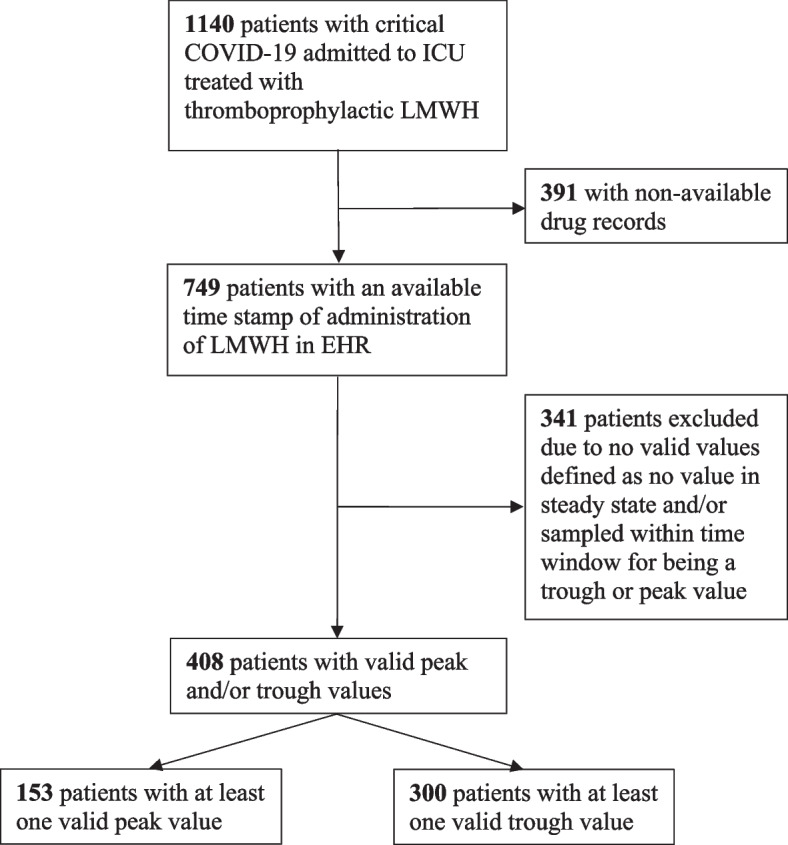
Flow chart. Patients with critical COVID-19 and valid peak and trough values of anti-Factor Xa

A total of 1,140 patients with critical COVID-19, with 7,302 collected aFXa values, were admitted from March 2020 to May 2021 to ICUs at the participating hospitals (Table 1). Three hundred ninety-one patients were cared for when ICUs had to expand beyond the capacity of PDMS systems, and drugs were temporarily registered on paper records. Since it was not possible to collect time stamps of LMWH administration from paper records, these patients were excluded. Of the remaining 749 patients, 408 had at least one valid aFXa value according to the predefined requirements on steady state and time between administration of LMWH and blood sampling. This resulted in a peak cohort of 153 patients with a total of 335 peak values and a trough cohort of 300 patients with a total of 766 trough values (Fig. 1). Forty-five patients had both peak and trough values and were therefore included in both cohorts. Baseline characteristics displayed in Table 1.

**Table ﻿1:** Baseline characteristics of patients with critical COVID-19 admitted from March 2020 to May 2021 to ICUs at the participating hospitals with no diagnosis of TE or major bleeding at admission

**Characteristic**	All patients (*n* = 1140)	Patients with peak values (*n* = 153)	Patients with trough value (*n* = 300)
**Hospital**			
Södersjukhuset	625 (54.8)	57 (37.3)	88 (29.3)
Karolinska University Hospital	426 (37.3)	47 (30.7)	209 (69.7)
Skåne University Hospital	89 (7.8)	49 (32.0)	3 (1.0)
**Age,** median (IQR), years	63 (54 to 71)	64 (55 to 70)	63 (54 to 69)
**Sex**			
Male	840 (73.7)	128 (83.7)	225 (75.0)
Female	300 (26.3)	25 (16.3)	75 (25.0)
BMI, median (IQR), kg/m^2^	(*n* = 1109) 29 (26 to 33)	(*n* = 144) 29 (26 to 34)	(*n* = 296) 29 (26 to 35)
**Coexisting conditions**			
Hypertension	576 (50.5)	77 (50.3)	159 (53.0)
Diabetes mellitus	285 (25.0)	42 (27.4)	74 (24.7)
Heart disease (IHD, Heart failure, Atrial fibrillation/flutter)	194 (17.0)	22 (14.4)	49 (16.3)
Chronic pulmonary disease	266 (23.3)	29 (19.0)	73 (24.3)
Immunosuppressive	77 (6.8)	10 (6.5)	30 (10.0)
Prior venous thromboembolic disease (no longer treated)	51 (4.5)	2 (1.3)	14 (4.7)
Prior cerebrovascular disease	82 (7.2)	8 (5.2)	22 (7.3)
Renal disease	95 (8.3)	11 (7.2)	28 (9.3)
Liver disease	38 (3.3)	5 (3.3)	13 (4.3)
Malignancy	113 (9.9)	9 (5.9)	35 (11.7)
**Chronic use of medication**			
Systemic glucocorticoids	84 (7.4)	13 (8.5)	24 (8.0)
Direct oral anticoagulants	78 (6.8)	5 (3.3)	19 (6.3)
Vitamin K antagonists	13 (1.1)	0 (0)	3 (1.0)
Platelet inhibitors	153 (13.4)	24 (15.7)	35 (11.7)
SAPS III- score, median (IQR)	(*n* = 1086) 56 (51 to 63)	(*n* = 142) 58 (53 to 64)	(*n* = 280) 57 (50 to 62)
PaO2/FiO2 at admission, median (IQR)	(*n* = 966) 11 (9.0 to 14)	(*n* = 97) 11 (8.9 to 14)	(*n* = 273) 11 (8.6 to 13)
Limitations of care (life support or CPR)	83 (7.3)	4 (2.6)	15 (5.0)
Time from onset of symptoms to ICU admission, median (IQR), days	(*n* = 1139) 10 (7 to 13)	9 (7 to 12)	10 (7 to 12)
**Respiratory support during current admission**			
High flow nasal cannula	(*n* = 1051) 572 (54.4)	(*n* = 104) 48 (46)	(*n* = 297) 124 (41.8)
Noninvasive ventilation	(*n* = 1051) 426 (40.5)	(*n* = 104) 27 (26)	(*n* = 297) 161 (54.2)
Invasive ventilation	735 (64.5)	145 (95)	227 (75.7)
**Therapy during current admission**			
Glucocorticoids	942 (82.6)	126 (82.4)	293 (97.7)
IL-6 inhibitor	158 (13.9)	11 (7.2)	69 (23.0)
Remdesivir	137 (12.0)	13 (8.5)	55 (18.3)
Platelet inhibitors	(*n* = 815) 153 (18.8)	(*n* = 63) 24 (38.1)	(*n* = 265) 67 (25.3)
**Initial dose of LMWH**^**a**^			
High LMWH dose^b^	(*n* = 1100) 299 (27.2)	(*n* = 147) 22 (15.0)	(*n* = 288) 71 (24.7)
Intermediate LMWH dose^c^	(*n* = 1100) 636 (57.8)	(*n* = 147) 95 (64.6)	(*n* = 288) 194 (67.4)
Low LMWH dose^d^	(*n* = 1100) 142 (12.9)	(*n* = 147) 30 (19.6)	(*n* = 288) 22 (7.6)
No prophylaxis	(*n* = 1100) 18 (1.6)	(*n* = 147) 0 (0)	(*n* = 288) 1 (0.3)
Other drug than LMWH	(*n* = 1100) 5 (0.5)	(*n* = 147) 0	(*n* = 147) 0
**Type of LMWH**			
Tinzaparin	(*n* = 1010) 558 (55.2)	56 (36.6)	81 (27.0)
Dalteparin	(*n* = 1010) 367 (36.3)	48 (31.4)	216 (72.0)
Enoxaparin	(*n* = 1010) 85 (8.4)	49 (32.0)	3 (1.0)
**Laboratory markers**^**e**^			
Hemoglobin, median (IQR), g/L	(*n* = 1033) 131 (119 to 141)	(*n* = 140) 133 (121 to 147)	(*n* = 281) 132 (119 to 141)
Leucocyte count, median (IQR), 10^9/L	(*n* = 995) 8.8 (6.5 to 11.8)	(*n* = 136) 8.8 (6.0 to 12)	(*n* = 272) 8.5 (6.3 to 11)
Platelet count, median (IQR), 10^9/L	(*n* = 994) 238 (182 to 307)	(*n* = 136) 235 (178 to 311)	(*n* = 271) 225 (178 to 283)
Activated partial thrombin time, median (IQR), seconds	(*n* = 720) 27 (24 to 30)	(*n* = 99) 28 (25 to 31)	(*n* = 251) 26 (24 to 30)
Prothrombin time, median (IQR), INR	(*n* = 959) 1.1 (1 to 1.2)	(*n* = 133) 1.1 (1 to 1.2)	(*n* = 267) 1.1 (1 to 1.2)
Fibrin-D-dimer, median (IQR), mg/L FEU	(*n* = 906) 1.2 (0.8 to 2.4)	(*n* = 126) 1.5 (0.8 to 2.8)	(*n* = 264) 1.2 (0.7 to 2.3)
CRP, median (IQR), mg/L	(*n* = 994) 148 (92 to 224)	(*n* = 153) 154 (100 to 236)	(*n* = 272) 145 (87 to 217)
Procalcitonin, median (IQR), µg/L	(*n* = 904) 0.36 (0.18 to 0.97)	(*n* = 93) 0.46 (0.23 to 1.2)	(*n* = 263) 0.38 (0.17 to 1.2)
Bilirubin, median (IQR), µmol/L	(*n* = 976) 8 (5 to 11)	(*n* = 133) 8 (6 to 11)	(*n* = 269) 7 (5 to 11)
Fibrinogen, median (IQR), g/L	(*n* = 706) 6.3 (5.1 to 7.4)	(*n* = 92) 6.5 (5.3 to 7.7)	(*n* = 250) 6.0 (4.9 to 7.2)
Creatinine, median (IQR), µmol/L	(*n* = 1032) 70 (57 to 92)	(*n* = 152) 75 (60 to 100)	(*n* = 299) 71 (58 to 93)
eGFR by CKDEpi, median (IQR), ml/min/1.73 m^2^	(*n* = 1032) 97 (76 to 107)	(*n* = 152) 95 (70 to 106)	(*n* = 299) 96 (73 to 107)
Patients with eGFR below 30 by CKDEpi, ml/min/1.73 m^2^	(*n* = 1032) 58 (5.6)	(*n* = 152) 12 (7.9)	(*n* = 299) 20 (6.7)
Patients with eGFR below 60 by CKDEpi, ml/min/1.73 m^2^	(*n* = 1032) 172 (16.7)	(*n* = 152) 28 (18.4)	(*n* = 299) 50 (16.7)
Patients with eGFR below 90 by CKDEpi, ml/min/1.73 m^2^	(*n* = 1032) 393 (38.1)	(*n* = 152) 62 (40.5)	(*n* = 299) 116 (38.7)

Baseline characteristics of 1,140 patients, admitted to the ICU from March 2020 to May 2021 due to critical COVID-19

Values are expressed as no. (%) unless otherwise indicated. Data are complete for all included patients unless indicated by the number of patients

*Abbreviations*: *BMI* body mass index, *IHD* ischemic heart disease, *SAPS III* Simplified Acute Physiology Score III, *PaO2/FiO2* partial pressure of oxygen/fraction of inspired oxygen, *IL-6* interleukin-6, *LMWH* low-molecular-weight heparin, *INR* international normalized ratio, *FEU* fibrinogen equivalent unit, *CRP* C-reactive protein, *CPR* cardiopulmonary resuscitation, *eGFR* estimated glomerular filtration rate, *CKDEpi* Chronic Kidney Disease Epidemiology Collaboration, *ICU* intensive care unit, *aFXa* anti-Factor Xa

^a^At ICU admission defined as the first date during the ICU stay

^b^Tinzaparin, ≥ 175 IU/kg of body weight per daily, dalteparin, ≥ 200 IU/kg of body weight daily, or enoxaparin, ≥ 2 mg/kg of body weight daily

^c^Tinzaparin, > 4500 IU daily to < 175 IU/kg of body weight daily, or dalteparin, > 5000 IU daily to < 200 IU/kg of body weight daily, or enoxaparin, > 40 mg but < 2 mg/kg of body weight daily

^d^Tinzaparin, 2500–4500 IU daily, dalteparin, 2500–5000 IU daily, or enoxaparin, ≤ 40 mg daily

^e^Baseline laboratory values defined as those sampled within 48 h of ICU admission

### aFXa

The number of valid values per patient, the minimum value during the ICU stay, the median value during the first 14 ICU days, and the maximum value during the ICU stay are displayed in Table 2.

**Table 2:** aFXa and outcomes for patients with critical COVID-19 admitted from March 2020 to May 2021 to ICUs at the participating hospitals with no diagnosis of TE or major bleed at admission

	All patients (*n* = 1140)	Patients with peak values (*n* = 153)	Patients with trough values (*n* = 300)
No of aFXa during ICU stay per patient, median (IQR)	-	1 (1 to 3)	2 (1 to 3)
aFXa, minimum value during ICU stay, median (IQR), kIU/L	-	0.41 (0.29 to 0.55)	0.21 (0.14 to 0.34)
aFXa, median value over the first 14 ICU days, median (IQR), kIU/L	-	(*n* = 126) 0.5 (0.34 to 0.64)	(*n* = 266) 0.29 (0.21 to 0.46)
aFXa, maximum value during ICU stay, median (IQR), kIU/L	-	0.55 (0.39 to 0.76)	0.38 (0.24 to 0.56)
Days in ICU, median (IQR)	11 (5 to 22)	19 (13 to 30)	17 (9 to 28)
Death within 90 days	314 (27.5)	55 (35.9)	95 (31.7)
Death in ICU	258 (22.6)	49 (32.0)	88 (29.3)
Any thromboembolism and/or bleeding	378 (33.2)	57 (37.3)	117 (39.0)
Thromboembolism	160 (14.0)	17 (11.1)	37 (12.3)
Pulmonary embolism/thrombosis^a^	134 (11.8)	14 (9.2)	31 (10.3)
Deep venous thrombosis^b^	32 (2.8)	3 (2.0)	5 (1.7)
Ischemic stroke^a^	11 (1.0)	2 (1.3)	3 (1.0)
Bleeding^c^	288 (25.2)	48 (31.3)	100 (33.3)
Major bleeding^d^	72 (6.3)	15 (9.8)	23 (7.7)

Outcome of 1,140 patients admitted to the ICU due to critical COVID-19

Values are expressed as no. (%) unless otherwise indicated. Data are complete for all included patients unless indicated by the number of patients

*Abbreviations*: *aFXa* anti-Factor Xa, *ICU* intensive care unit

^a^Defined as a diagnosis verified by computed tomography

^b^Defined as a diagnosis verified by ultrasound or computed tomography

^c^Defined as World Health Organization bleeding scale grade 1–4

^d^Defined as World Health Organization bleeding scale grade 3–4

### Outcomes

Outcomes for all patients, including patients without valid aFXa values, are displayed in Table 2. Patients with peak values had a median ICU length of stay of 19 days (IQR 13–30), and patients with trough values had a median ICU length of stay of 17 days (IQR 9 to 22). The incidences of death, TE, bleeding, and major bleeding were 35.9%, 11.1%, 31.3%, and 9.8% for patients with peak values and 31.7%, 12.3%, 33.3%, and 7.7% for patients with trough values, respectively. Most patients who died within 90 days, died in the ICU (Table 2). Bleeding, but not TE, was associated with an increased risk of death (Supplement, Table S1). The number of valid aFXa values by peak and trough group and by event of TE, bleeding, and/or major bleeding is shown in Table 2 and Supplement, Table S2, respectively.

### Association between peak values and outcome

**Fig. 2:**
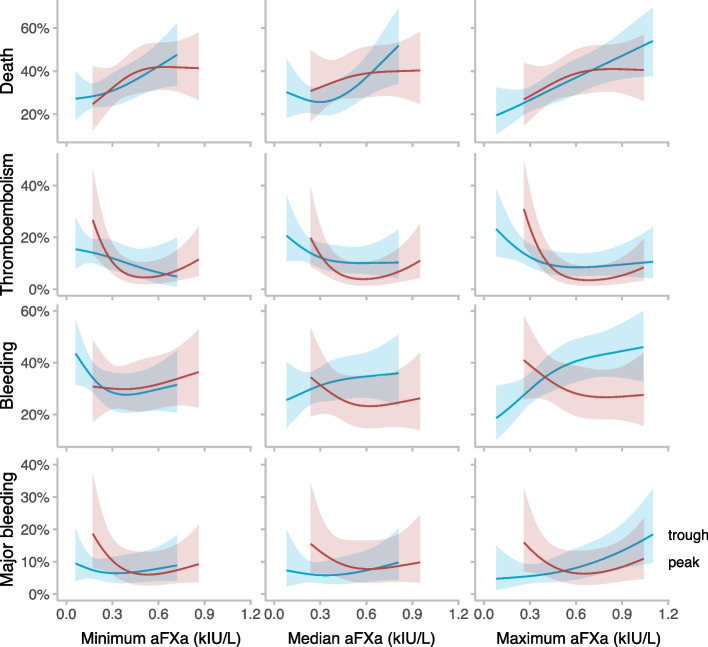
The association between anti-Factor Xa and death, thromboembolism, bleeding and major bleeding. Red lines represent peak values, blue lines represent trough values, and the shaded area represents 95% confidence intervals. The figures illustrate anti-Factor Xa values when summarized as minimum values during intensive care (153 peak values and 300 trough values), median values during the first 14 days of intensive care (126 peak values and 266 trough values), and maximum values during intensive care (153 peak values and 300 trough values). For peak values, minimum, median and maximum values were not associated with death (*p* = 0.33, 0.75 and 0.44). The minimum, median and maximum values were all associated with thromboembolism (*p* = 0.005, 0.01 and 0.001). Minimum, median and maximum anti-Factor Xa were not associated with bleeding (*p* = 0.74, 0.60 and 0.41) or major bleeding (*p* = 0.19, 0.57 and 0.22). For trough values, not minimum but median and maximum values were associated with death (*p* = 0.05, 0.03 and 0.002). The minimum, median and maximum values were not associated with thromboembolism (*p* = 0.31, 0.31 and 0.10). Not the minimum and median but the maximum value was associated with bleeding (*p* = 0.19, 0.57 and 0.01) or major bleeding (*p* = 0.63, 0.46 and 0.02)

Figure 2 displays the nature of the relationship of all outcomes and peak values. When peak values were visualized against death, no association was seen with either patients’ minimum, median, or maximum value. However, lower minimum, median and maximum peak values were all associated with a higher risk of TE (*p* = 0.005, 0.01, and 0.001). When investigating different cut-offs, patients in the group with a minimum value below 0.3 kIU/L had an OR of 5.1 (95% CI 1.8 to 14.4) for TE compared to the patients with no value below 0.3 kIU/L (Supplement, Table S3a), with the distribution visualized in Supplement, Fig. S1. Peak values had no association with bleeding or major bleeding, and no cut-off value could separate patients into groups with different risks (Supplement Table S3b and Fig. S1).

### Association between trough values and outcome

The association between trough values and outcome is displayed in Fig. 2. Not for minimum but for median and maximum, increasing trough values were associated with death (*p* = 0.03 and 0.002). Median values above 0.5 kIU/L (OR 1.9, 95% CI 1.0 to 3.5) and maximum values above 0.3 kIU/L (OR 1.73, 95% CI 1.0 to 3.0) were identified as significant cut-offs for an increased risk (Table S3c-d and Fig. S1). Trough values were not associated with TE, and no significant cut-off values were found (Supplement, Table S3e and Fig. S1). Maximum values were significantly associated with bleeding and major bleeding, *p* = 0.01 and 0.02, with a constant increasing risk with higher trough values. An increased risk of bleeding was seen with maximum values above 0.3 kIU/L (OR 1.9, 95% CI 1.1 to 3.3) and an increased risk of major bleeding with maximum values above 0.5 kIU/L (OR 2.4, 95% CI 1.0 to 5.6) compared to patients with no value above these cut-offs (Supplement, Table S3f and Fig. S1). Additionally, for bleeding and major bleeding, higher cut-offs than 0.3 and 0.5 kIU/L could separate the cohort into groups with different risks.

### Renal function

The correlation between aFXa and eGFR is displayed in Supplement, Fig. S2. For peak values (minimum, median and maximum), no significant correlation with eGFR was found. For trough values, there were modest but significant correlations with eGFR for median and maximum values, with a lower eGFR resulting in a higher value of aFXa. For visualization, eGFR values of 60, 90 and 120 ml/min/1.73m^2^ were used, as this best represented the spread of eGFR for the cohorts.

For peak values, the results in the more complex model including eGFR were unchanged, with lower minimum, median, and maximum values being associated with a higher risk of TE, *p* = 0.01, 0.01 and 0.001, respectively (Supplement, Fig. S3). Associations between trough values and outcomes when including eGFR were unaffected except for the association between higher maximum values and an increased risk of major bleeding, *p* = 0.06. Even though this association became nonsignificant in the more complex model, the curves retained the same shapes as the crude curves, indicating no change in the underlying relationship between aFXa and outcomes (Supplement, Fig. S4).

### LMWH

Baseline characteristics, aFXa values and outcomes by type of LMWH are displayed in Supplement, Table S4a-b, and the relationship between aFXa values by LMWH is visualized in Supplement, Fig. S5. With the exception of one patient in the peak cohort and seven patients in the trough cohort, the hospitals were perfectly separated by type of LMWH. The aFXa value achieved by different initial doses of LMWH can be seen in Supplement, Table S5.

## Discussion

In this retrospective, multicenter cohort study, we found an association of an increased risk of TE with lower aFXa peak values and an increased risk of death and bleeding with higher aFXa trough values in patients with critical COVID-19 treated with thromboprophylactic LMWH. To the best of our knowledge, this is the first report to show an association between aFXa values and outcomes in patients with critical COVID-19 and therefore hypothesis-generating for future studies investigating aFXa-guided LMWH prophylaxis.

For peak values, the risk for TE steeply increased below values of approximately 0.3 kIU/L, indicating the importance of achieving peak levels above this cut-off to counteract the procoagulative condition in patients with critical COVID-19. This also suggests that peak values are preferred when monitoring the effect of LMWH in patients where TE could have detrimental effects, for example, in patients with heart failure or already limited gas exchange.

The association between a higher aFXa value and increased risk of death was only demonstrated with trough values and not with peak values. One possible reason for this association could be that more severe organ dysfunction, including renal failure, causes both drug accumulation and a higher risk of death. Another explanation may be the association with bleeding events in patients with higher trough values, since major bleedings have previously been associated with increased mortality in patients with critical COVID-19 [27–30]. These results are in line with our data, as patients with bleeding events also had an increased risk of death.

In the present study, the maximum trough value during the ICU stay was associated with an increased number of bleedings. Values above 0.3 kIU/L and 0.5 kIU/L doubled the odds of bleeding and major bleeding, respectively. This suggests that one value above these cut-offs could be enough to justify increased surveillance for potential bleeding events. Accordingly, for patients with a high risk of bleeding, for example, patients newly exposed to surgery or other invasive procedures, trough values could be preferred for monitoring.

aFXa values in COVID-19 patients were previously investigated by Hamilton et al., but they found no relationship between aFXa and outcomes [31]. However, compared to our study, the study by Hamilton et al. was smaller, with 58 patients from a single center setting and only two patients developing TE. Monitoring anticoagulation in patients with critical COVID-19 has also been studied by Bunch et al. They found a significant reduction in bleeding when using a dosing protocol based on viscoelastic testing, indicating that monitoring is important for COVID-19 patients, as hypercoagulation may vary with disease severity and during the disease course [32].

Renal dysfunction can affect aFXa due to changes in pharmacokinetics but can also be an independent risk factor for death. Therefore, we added eGFR as a confounder to our model. For one out of 24 associations, the result of Wald ´s test changed. This was a result of a significant association in the crude analysis where the p values increased slightly above the predefined limit of 0.05. This was expected, as the number of patients and the event rates were predicted to be too small for a more complex model. However, the wide CI indicates possible variability in outcome.

We did demonstrate a relationship between patients categorized by initial dose of LMWH and trough values as illustrated in Supplement, Table S5. However, it should be noted that the relationship was not seen for peak values or for all categorize of trough values. The reason for this may be a statistical type 2 error, as the number of patients in low and high dose LMWH were limited. This could also be because the dose of LMWH changed during ICU care, or because other variables than just the dose of LMWH affects what aFXa value that will be achieved.

We recognize the limitations of the present study. First, given its retrospective nature, causality between the classified levels of aFXa and outcomes cannot be established. Second, due to missing data, especially administration time stamps for LMWH, the number of patients with valid aFXa values was lower than expected. This affected the power of the study and the number of complex analyses that could be performed. The limited number of valid values was partly due to the strict definition of peak and trough values. However, this strict definition was also a strength in the present study and led to the discovery of associations between aFXa and clinical outcomes that previously have not been established. Third, compared to the whole cohort, patients with valid aFXa values had a longer ICU stay and a higher mortality, which might introduce a selection bias and therefore may impact generalizability. Fourth, we did not adjust for multiple testing for the primary outcome, as the aim of this retrospective study was exploratory, and we wanted to minimize the risk of not detecting true associations and differences (Type 2 statistical error). Therefore, all significant associations and differences must be interpreted carefully. Fifth, the investigation of TE was not performed by screening but rather at the discretion of the treating clinician, and the risk of underdiagnosing of the outcome must be considered. Sixth, heterogeneity in aFXa monitoring might have introduced bias. Although two of the three hospitals had a routine for aFXa monitoring, it cannot be excluded that a significant proportion of the aFXa values were sampled from patients with a higher risk of TE and/or bleeding compared to the population in whole.

## Conclusion

Measuring aFXa activity may be relevant when administering LMWH to patients with critical COVID-19. Lower peak values were associated with an increased risk of TE and higher trough values were associated with an increased risk of death and bleeding. Prospective studies are needed to confirm the results.

### Supplementary Information

**Additional file 1.** Local treatment guidelines, Tables S1 to S5, and Fig. S1 to S5. **Local treatment guidelines **from Södersjukhuset, Karolinska University Hospital, and Skåne University Hospital. **Table S1.** Risk of death within 90 days if suffering a TE, bleeding, or major bleeding within 28 days compared to no event. **Table S2.** Number of values of aFXa by event of thromboembolism, bleeding, and/or major bleeding. **Table S3a-f.** Risk for thromboembolism, bleeding, major bleeding and death by different anti-Factor Xa cut-off values. **Table S4a-b.** Baseline characteristics, anti-Factor Xa values, and outcomes by type of low-molecular-weight heparin. **Table S5a-b.** Values of aFXa by initial dose of low-molecular-weight heparin. **Fig. S1.** Distribution of patients’ minimum and maximum anti-Factor Xa values during intensive care stay with proportions of outcomes of thromboembolism, bleeding, major bleeding, and death. **Fig. S2.** Scatterplot of correlation between anti-Factor Xa values and estimated glomerular filtration rate. **Fig. S3.** Anti-Factor Xa peak values and association with outcomes adjusted for estimated glomerular filtration rate. **Fig. S4.** Anti-Factor Xa trough values and association with outcome adjusted for estimated glomerular filtration rate. **Fig. S5.** Distribution of anti-Factor Xa values by type of low-molecular-weight heparin.

## Data Availability

The datasets analysed during the current study are not publicly available due to privacy or ethical restrictions but are available on reasonable request from the corresponding author.

## Abbreviations

CKD-Epi: Chronic Kidney Disease Epidemiology Collaboration
CI: Confidence interval
DVT: Deep venous thrombosis
eGFR: Estimated glomerular filtration rate
EHR: Electronic health record
ICU: Intensive care unit
IQR: Interquartile range
IU: International units
LMWH: Low-molecular-weight heparin
OR: Odds ratio
PDMS: Patient data management system
PE/PT: Pulmonary embolism/thrombosis
TE: Thromboembolism
