# Supplementary material for: A retrospective multicenter cohort study of the association between anti-Factor Xa values and death, thromboembolism, and bleeding in patients with critical COVID-19
Source: Thromb J. 2023 Oct 2;21:101. doi: 10.1186/s12959-023-00541-z (PMC10544466; doi:10.1186/s12959-023-00541-z)
Supplement: Supplementary file 1 — Additional file 1. Local treatment guidelines, Tables S1 to S5, and Fig. S1 to S5. Local treatment guidelines from Södersjukhuset, Karolinska University Hospital, and Skåne University Hospital. Table S1. Risk of death within 90 days if suffering a TE, bleeding, or major bleeding within 28 days compared to no event. Table S2. Number of values of aFXa by event of thromboembolism, bleeding, and/or major bleeding. Table S3a-f. Risk for thromboembolism, bleeding, major bleeding and death by different anti-Factor Xa cut-off values. Table S4a-b. Baseline characteristics, anti-Factor Xa values, and outcomes by type of low-molecular-weight heparin. Table S5a-b. Values of aFXa by initial dose of low-molecular-weight heparin. Fig. S1. Distribution of patients’ minimum and maximum anti-Factor Xa values during intensive care stay with proportions of outcomes of thromboembolism, bleeding, major bleeding, and death. Fig. S2. Scatterplot of correlation between anti-Factor Xa values and estimated glomerular filtration rate. Fig. S3. Anti-Factor Xa peak values and association with outcomes adjusted for estimated glomerular filtration rate. Fig. S4. Anti-Factor Xa trough values and association with outcome adjusted for estimated glomerular filtration rate. Fig. S5. Distribution of anti-Factor Xa values by type of low-molecular-weight heparin. [file 12959_2023_541_MOESM1_ESM.docx]

**Supplement**

**A retrospective multicenter cohort study of the association between anti-Factor Xa values and death, thromboembolism, and bleeding in patients with critical COVID-19**

Table of Contents

[Local treatment guidelines 2](#_Toc145269461)

[Table S1. Risk of death within 90 days by event of TE, bleeding, and/or major bleeding within 28 days compared to no event. 6](#_Toc145269462)

[Table S2: Number of values of anti-Factor Xa by event of thromboembolism, bleeding, and/or major bleeding. 7](#_Toc145269463)

[Table S3a-f. Risk for thromboembolism, bleeding, major bleeding and death by different anti-Factor Xa cut-off values. 8](#_Toc145269464)

[Table S4a-b. Baseline characteristics, anti-Factor Xa values, and outcomes by type of low-molecular-weight heparin. 10](#_Toc145269465)

[Table S5a-b: Values of anti-Factor Xa by initial dose of low-molecular-weight heparin 12](#_Toc145269466)

[Fig. S1. Distribution of patients’ minimum and maximum anti-Factor Xa values during intensive care stay with proportions of outcomes of thromboembolism, bleeding, major bleeding, and death. 13](#_Toc145269467)

[Fig. S2. Scatterplot of the correlation between anti-Factor Xa values and estimated glomerular filtration rate. 14](#_Toc145269468)

[Fig. S3. Anti-Factor Xa peak values and association with outcomes adjusted for estimated glomerular filtration rate. 15](#_Toc145269469)

[Fig. S4. Anti-Factor Xa trough values and association with outcome adjusted for estimated glomerular filtration rate. 16](#_Toc145269470)

[Fig. S5. Distribution of anti-Factor Xa values by type of low-molecular-weight heparin. 17](#_Toc145269471)

## Local treatment guidelines

This document contains guidelines concerning thromboprophylaxis for patients with critical COVID-19 at our ICUs. They have been translated from Swedish to English.

**Anesthesia and Intensive care**

| Administrative dept: | | Unit: |
| --- | --- | --- |
| Dept of Anaesthesia and intensive care | | **Surgical ICU** |
| Document type: | | Document no: |
| **Guideline** | | 000474 |
| Document name: | | |
| **Covid-19 – airways, intubation, and treatment. Patients in ICU with suspicious or confirmed infection** | | |
| Created | Developed by: | |
| 032720 | Marianne Mörrby Ramberg, Brian Cleaver | |
| Last revision: | Revised by: | |
| 041620 | Marianne Mörrby Ramberg, Wolfram Johnen | |
| Printed on: | Approved by dept chief | |
| 041620 | Emma Jerkegren-Olsson | |

| **[…]** 200327: Thromboprophylaxis with Innohep® (tinzaparin), 4500 IU once daily, to patients with no contraindication.  Revision 200408: Patients with COVID-19 appear to have an increased risk for thromboembolic complications. Therefore, thromboprophylaxis dose should be given twice daily to patients with no contraindications.  Revision 200416: Due to high frequency of thromboembolic complications among critically ill COVID-19 patients we have decided to increase the dose of thromboprophylaxis. There is no strong evidence for this treatment decision, continuous evaluation will be performed and the treatment regime may change. If no contraindications or tendency of bleeding exists critically ill COVID-19 patients will be prescribed Innohep® (tinzaparin), 100 IU/kg twice daily. **[…]** |
| --- |


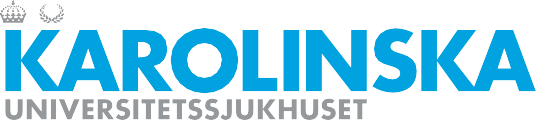


| Author:  Anders Oldner, Lars I Eriksson, Christina Agvald-Öhman | Pages: 7 |
| --- | --- |
|  | Doc-ID: Kar2-17857 |
| Approved by:  Björn Persson/Karolinska/SLL | Created: April 2020 |
|  | Valid thru: 2021-07-09 |

Function PMI – Perioperative Medicine and Intensive care

Pharmacological treatment of COVID-19 in intensive care units

**[…]**

Currently, there is no firm evidence-based foundation to guide thromboprophylaxis dosage for critically ill COVID-19 patients. Until more evidence or information regarding treatment strategies is made available, the following doses are recommended for **thromboprophylaxis**:

Weight:

50-90 kg Fragmin® (dalteparin) 5000 IU twice daily, or equivalent

<50 kg Fragmin® (dalteparin) 2500 IU twice daily, or equivalent

>90 kg Fragmin® (dalteparin) 7500 IU twice daily, or equivalent

The dose may need adjustment with regard to the degree of illness and tendency to bleed.

**[…]**

| Headline  **Covid19, coagulation**  Author: **Wallquist, Wilhelm** | Document type **Guideline** Approved by **Bonnevier Johan** | Created: **2020-05-11** | Valid thru: **2022-05-10** |
| --- | --- | --- | --- |


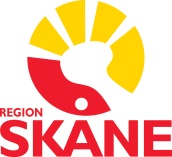


**Covid-19, coagulation guideline for patients treated in the intensive care**

**[…]**

Thromboprophylaxis should be prescribed to all patients treated for critical COVID-19 if no contraindications are present (allergies, history of HIT, platelets < 30 * 10^9/L, ongoing or a high risk of bleeding).

Weight < 50 kg Klexane® (enoxaparin) 20 mg twice daily or 40 mg once daily

Weight 50-90 kg Klexane® (enoxaparin) 40 mg twice daily

Weight > 90 kg Klexane® (enoxaparin) 60 mg twice daily

For patients with renal dysfunction (creatinine clearance <30 ml/min), half the dose should be prescribed.

**[…]**

## **Table S1.** Risk of death within 90 days by event of TE, bleeding, and/or major bleeding within 28 days compared to no event.

**Table S1:** Risk of death by event of TE, bleeding, and/or major bleeding.

|  | Odds ratios (95% CI) of death by event of TE, bleeding, or major bleeding compared to no event | | |
| --- | --- | --- | --- |
|  | All patients (n = 1140) | Patients with peak values (n = 153) | Patients with trough value (n = 300) |
| Thromboembolism | 1.2 (0.83 to 1.7) | 0.51 (0.16 to 1.66) | 1.0 (0.50 to 2.2) |
| Bleeding | 2.2 (1.6 to 2.9)^a^ | 2.7 (1.3 to 5.5)^c^ | 2.5 (1.5 to 4.1)^d^ |
| Major bleeding | 2.4 (1.5 to 3.8)^b^ | 1.2 (0.41 to 3.6) | 2.1 (0.89 to 5.0) |

^a^ *p* < 0.001

^b^ *p* < 0.001

^c^ *p* = 0.005

^d^ *p* < 0.001

## **Table S2**: Number of values of anti-Factor Xa by event of thromboembolism, bleeding, and/or major bleeding.

|  | Patients with no TE, bleeding, or major bleeding | Patients with TE, bleeding, and/or major bleeding^a^ | *p* value^b^ |
| --- | --- | --- | --- |
| No of aFXa during ICU stay per patient in the peak group, median (IQR) | 2 (1 to 3) | 1 (1 to 2) | <0.001 |
| No of aFXa during ICU stay per patient in the trough group, median (IQR) | 1 (1 to 3) | 2 (1 to 4) | <0.001 |

^a^ Values after event not included

^b^ *p* values for differences across groups were obtained using the **Mann-Whitney test**

## **Table S3a-f.** Risk for thromboembolism, bleeding, major bleeding and death by different anti-Factor Xa cut-off values.

**Table S3a:** Minimum peak value and risk of thromboembolism

| aFXa-cut-off (kIU/L) | Patients ever below out of 153, no | Odds ratios of thromboembolism if ever below cut-off compared to no value below (95 % CI) |
| --- | --- | --- |
| 0.1 | 0 | ^a^ |
| 0.2 | 15 | 3.5 (0.97 to 12.6) |
| 0.3 | 40 | 5.1 (1.8 to 14.4)^b^ |
| 0.4 | 71 | 2.3 (0.81 to 6.6) |
| 0.5 | 98 | 2.0 (0.60 to 6.3) |
| 0.6 | 124 | 0.73 (0.22 to 2.4) |
| 0.7 | 133 | 0.67 (0.17 to 2.6) |

Odds ratios for different cut-off values of minimum peak aFXa for 153 patients admitted to the ICU due to critical COVID-19.

^a^ No patients in one group

*^b^* *p* = 0.003

**Table S3b:** Maximum peak value and risk of bleeding and major bleeding

| afXa-cut-off (kIU/L) | Patients ever above to out of 153, no | Odds ratio of bleeding if ever above cut-off compared to no value above (95 % CI) | Odds ratio of major bleeding if ever above cut-off compared to no value above (95 % CI) |
| --- | --- | --- | --- |
| 0.1 | 152 | ^a^ | ^a^ |
| 0.2 | 148 | 1.9 (0.20 to 17.1) | ^a^ |
| 0.3 | 136 | 0.47 (0.17 to 1.3) | 0.29 (0.08 to 1.0) |
| 0.4 | 110 | 0.52 (0.25 to 1.1) | 0.55 (0.18 to 1.7) |
| 0.5 | 90 | 0.67 (0.34 to 1.3) | 0.58 (0.20 to 1.7) |
| 0.6 | 63 | 0.80 (0.40 to 1.6) | 0.69 (0.22 to 2.1) |
| 0.7 | 43 | 0.57 (0.26 to 1.3) | 0.61 (0.16 to 2.3) |

Odds ratios for different cut-off values of maximum peak aFXa for 153 patients admitted to the ICU due to critical COVID-19.

^a^ No outcomes in one group

| aFXa-cut-off (kIU/L) | Patients below out of 266, no | Odds ratio of death if below cut-off compared to equal or above (95 % CI) | Patients above out of 266, no | Odds ratio of death if above cut-off compared to below or equal (95 % CI) |
| --- | --- | --- | --- | --- |
| 0.1 | 15 | 1.15 (0.38 to 3.48) | 247 | 0.94 (0.35 to 2.58) |
| 0.2 | 57 | 0.86 (0.45 to 1.65) | 202 | 1.42 (0.75 to 2.69) |
| 0.3 | 134 | 0.76 (0.45 to 1.29) | 128 | 1.43 (0.85 to 2.42) |
| 0.4 | 175 | 0.62 (0.36 to 1.06) | 86 | 1.47 (0.85 to 2.53) |
| 0.5 | 210 | 0.55 (0.30 to 1.01) | 55 | 1.90 (1.02 to 3.51)^a^ |
| 0.6 | 235 | 0.31 (0.14 to 0.66)^b^ | 31 | 3.24 (1.51 to 6.96)^c^ |
| 0.7 | 246 | 0.41 (0.16 to 1.02)^d^ | 18 | 3.12 (1.18 to 8.22)^e^ |

**Table S3c:** Median trough value and risk of death

Odds ratios for different cut-off values of median trough aFXa for 266 patients admitted to the ICU due to critical COVID-19.

^a^ *p* = 0.04

^b^ *p* = 0.002

^c^ *p* = 0.002

^d^ *p* = 0.048

^e^ *p* = 0.017

| aFXa-cut-off (kIU/L) | Patients ever above out of 300, no | Odds ratio of death if ever above cut-off compared to no value above (95 % CI) |
| --- | --- | --- |
| 0.1 | 283 | 2.25 (0.63 to 8.02) |
| 0.2 | 246 | 2.03 (0.99 to 4.13) |
| 0.3 | 193 | 1.73 (1.02 to 2.95)^a^ |
| 0.4 | 139 | 1.64 (1.01 to 2.68)^b^ |
| 0.5 | 99 | 2.35 (1.42 to 3.91)^c^ |
| 0.6 | 62 | 2.96 (1.67 to 5.27)^d^ |
| 0.7 | 51 | 2.99 (1.62 to 5.55)^e^ |

**Table S3d:** Maximum trough value and risk of death

Odds ratios for different cut-off values of median trough aFXa for 300 patients admitted to the ICU due to critical COVID-19.

^a^ *p* = 0.041

^b^ *p* = 0.047

^c^ *p* = < 0.001

^d^ *p* = < 0.001

^e^ *p* = < 0.001

**Table S3e:** Minimum trough value and risk of thromboembolism

| aFXa-cut-off (kIU/L) | Patients ever below out of 300, no | Odds ratio of thromboembolism if ever below cut-off compared no value below cut-off (95 % CI) |
| --- | --- | --- |
| 0.1 | 36 | 1.9 (0.76 to 4.7) |
| 0.2 | 128 | 0.91 (0.45 to 1.8) |
| 0.3 | 207 | 1.5 (0.66 to 3.2) |
| 0.4 | 239 | 1.7 (0.64 to 4.7) |
| 0.5 | 260 | 6.3 (0.83 to 47.1) |
| 0.6 | 280 | 2.8 (0.36 to 21.6) |
| 0.7 | 287 | ^a^ |

Odds ratios for different cut-off values of minimum trough aFXa for 300 patients admitted to the ICU due to critical COVID-19.

^a^ No outcomes in one group

| aFXa-cut-off (kIU/L) | Patients ever above out of 300, no | Odds ratio of bleeding if ever above cut-off compared to no value above (95 % CI) | Odds ratio of major bleeding if ever above cut-off compared to no value above (95 % CI) |
| --- | --- | --- | --- |
| 0.1 | 283 | 1.7 (0.53 to 5.3) | 1.4 (0.17 to 10.7) |
| 0.2 | 246 | 1.5 (0.79 to 3.0) | 2.4 (0.55 to 10.7) |
| 0.3 | 193 | 1.9 (1.1 to 3.3)^a^ | 2.8 (0.93 to 8.5) |
| 0.4 | 139 | 1.9 (1.2 to 3.1)^b^ | 1.9 (0.79 to 4.5) |
| 0.5 | 99 | 2.1 (1.3 to 3.4)^c^ | 2.4 (1.01 to 5.6)^e^ |
| 0.6 | 62 | 2.1 (1.2 to 3.7)^d^ | 2.7 (1.1 to 6.6)^f^ |
| 0.7 | 51 | 1.7 (0.90 to 3.1) | 2.9 (1.2 to 7.3)^g^ |

**Table S3f:** Maximum trough value and risk of bleeding and major bleeding

Odds ratios for different cut-off values of maximum trough aFXa for 300 patients admitted to the ICU due to critical COVID-19.

^a^ *p* = 0.01

^b^ *p* = 0.009

^c^ *p* = 0.004

^d^ *p* = 0.01

^e^ *p* = 0.04

^f^ *p* = 0.05

^g^ *p*= 0.04

## **Table S4a-b.** Baseline characteristics, anti-Factor Xa values, and outcomes by type of low-molecular-weight heparin.

**Table S4a**. Peak values by different low-molecular-weight heparins

|  | Patients with tinzaparin (n = 56) | Patients with dalteparin (n = 48) | Patients with enoxaparin (n = 49) |
| --- | --- | --- | --- |
| **Hospital** |  |  |  |
| Södersjukhuset | 56 | 1 | 0 |
| Karolinska University Hospital | 0 | 47 | 0 |
| Skåne University Hospital | 0 | 0 | 49 |
| Age, median (IQR), years | 65 (53 to 72) | 59.5 (52 to 64) | 67 (56 to 73) |
| **Sex** |  |  |  |
| Male | 49 (87) | 42 (87) | 37 (75) |
| Female | 7 (13) | 6 (13) | 12 (25) |
| SAPS III- score, median (IQR) | 58 (53 to 64) | (n=37)  56 (52 to 60) | 59 (55 to 66) |
| Invasive ventilation | 49 (87) | 47 (98) | 49 (100) |
| **Initial dose of LMWH^a^** |  |  |  |
| High LMWH dose^b^ | 18 (32) | (n = 42)  1 (2.1) | 3 (6.1) |
| Intermediate LMWH dose^c^ | 34 (61) | (n = 42)  17 (40.5) | 44 (89.8) |
| Low LMWH dose^d^ | 4 (7.1) | (n = 42)  24 (57) | 2 (4.1) |
| No prophylaxis | 0 (0) | (n = 42)  0 (0) | 0 (0) |
| **Outcomes** |  |  |  |
| aFXa, minimum value during ICU stay, median (IQR), kIE/L | 0.40 (0.30 to 0.55) | 0.34 (0.27 to 0.56) | 0.47 (0.35 to 0.57) |
| aFXa, median value over the first 14 days after ICU admission, median (IQR), kIE/L | (n=44)  0.43 (0.33 to 0.60) | (n=38)  0.45 (0.32 to 0.62) | (n=44)  0.55 (0.44 to 0.68) |
| aFXa, maximum value during ICU stay, median (IQR), kIE/L | 0.49 (0.38 to 0.64) | 0.52 (0.32 to 0.67) | 0.68 (0.55 to 0.86) |
| Days in ICU, median (IQR) | 24 (16 to 30) | 21 (15 to 32) | 14 (10 to 24) |
| Death | 22 (39) | 15 (31) | 18 (37) |
| Thromboembolism | 3 (5.4) | 11 (23) | 3 (6.1) |
| Pulmonary embolism/thrombosis | 1 (1.8) | 10 (21) | 3 (6.1) |
| Deep venous thrombosis | 2 (3.6) | 1 (2.1) | 0 (0) |
| Ischemic stroke | 0 (0) | 2 (4.2) | 0 (0) |
| Bleeding | 20 (36) | 16 (33) | 12 (25) |
| Major bleeding | 4 (7.1) | 9 (19) | 2 (4.1) |

Baseline characteristics of 153 patients with peak values of aFXa admitted to the ICU due to critical COVID-19 by different low-molecular-weight heparins.

Values are expressed as no. (%) unless otherwise indicated. Data are complete for all included patients unless indicated by the number of patients.

Abbreviations: aFXa, anti-Factor Xa, SAPS III, Simplified Acute Physiology Score III, LMWH, low-molecular-weight heparin, ICU, intensive care unit

^a^ At ICU admission defined as the first date during the ICU stay.

^b^ Tinzaparin, ≥175 IU/kg of body weight per daily, dalteparin, ≥200 IU/kg of body weight daily, or enoxaparin, ≥2 mg/kg of body weight daily.

^c^ Tinzaparin, >4500 IU daily to <175 IU/kg of body weight daily, or dalteparin, >5000 IU daily to <200 IU/kg of body weight daily, or enoxaparin, ≥40 mg but <2 mg/kg of body weight daily.

^d^ Tinzaparin, 2500–4500 IU daily, dalteparin, 2500–5000 IU daily, or enoxaparin, <40 mg daily.

**Table S4b**: Trough values by different low-molecular-weight heparins

|  | Patients with tinzaparin (n= 81) | Patients with dalteparin (n=216) | Patients with enoxaparin (n=3) |
| --- | --- | --- | --- |
| **Hospital** |  |  |  |
| Södersjukhuset | 81 | 7 | 0 |
| Karolinska University Hospital | 0 | 209 | 0 |
| Skåne University Hospital | 0 | 0 | 3 |
| Age, median (IQR), years | 65 (52 to 72) | 63 (55 to 69) | 56 (47 to 57.5) |
| **Sex** |  |  |  |
| Male | 55 (68) | 168 (78) | 2 (67) |
| Female | 26 (32) | 48 (22) | 1 (33) |
| SAPS III- score, median (IQR) | 58 (53 to 64) | (n=196)  56 (50 to 62) | 56 (53 to 59) |
| Invasive ventilation | 58 (72) | 166 (77) | 3 (100) |
| **Initial dose of LMWH^a^** |  |  |  |
| High LMWH dose^b^ | 60 (74) | (n= 207)  11 (5.3) | 0 (0) |
| Intermediate LMWH dose^c^ | 20 (26) | (n= 207)  171 (83) | 3 (100) |
| Low LMWH dose^d^ | 0 (0) | (n= 207)  22 (11) | 0 (0) |
| No prophylaxis | 1 (1.2) | (n= 207)  0 (0) | 0 (0) |
| **Outcomes** |  |  |  |
| aFXa, minimum values during ICU stay, median (IQR), kIE/L | 0.31 (0.17 to 0.5) | 0.2 (0.13 to 0.29) | 0.34 (0.3 to 0.41) |
| aFXa, median values over the first 14 days after ICU admission, median (IQR), kIE/L | (n=75)  0.44 (0.27 to 0.56) | (n=188)  0.28 (0.19 to 0.38) | 0.34 (0.3 to 0.41) |
| aFXa, maximum values during ICU stay, median (IQR), kIE/L | 0.51 (0.37 to 0.62) | 0.33 (0.21 to 0.52) | 0.40 (0.37 to 0.44) |
| Days in ICU, median (IQR) | 16 (7 to 29) | 17 (9 to 27) | 17 (15 to 24) |
| Death | 30 (37) | 65 (30) | 0 (0) |
| Thromboembolism | 9 (11) | 27 (13) | 1 (33) |
| Pulmonary embolism/thrombosis | 8 (9.9) | 22 (10) | 1 (33) |
| Deep venous thrombosis | 1 (1.2) | 4 (1.9) | 0 (0) |
| Ischemic stroke | 1 (1.2) | 2 (1.0) | 0 (0) |
| Bleeding | 35 (43) | 64 (30) | 1 (33) |
| Major bleeding | 6 (7.4) | 17 (7.9) | 0 (0) |

Baseline characteristics of 300 patients with trough values of aFXa admitted to the ICU due to critical COVID-19 by different low-molecular-weight heparins.

Values are expressed as no. (%) unless otherwise indicated. Data are complete for all included patients unless indicated by the number of patients.

Abbreviations: aFXa, anti-Factor Xa, SAPS III, Simplified Acute Physiology Score III, LMWH, low-molecular-weight heparin, ICU, intensive care unit

^a^ At ICU admission defined as the first date during the ICU stay.

^b^ Tinzaparin, ≥175 IU/kg of body weight per daily, dalteparin, ≥200 IU/kg of body weight daily, or enoxaparin, ≥2 mg/kg of body weight daily.

^c^ Tinzaparin, >4500 IU daily to <175 IU/kg of body weight daily, or dalteparin, >5000 IU daily to <200 IU/kg of body weight daily, or enoxaparin, ≥40 mg but <2 mg/kg of body weight daily.

^d^ Tinzaparin, 2500–4500 IU daily, dalteparin, 2500–5000 IU daily, or enoxaparin, <40 mg daily.

## **Table S5a-b**: Values of anti-Factor Xa by initial dose of low-molecular-weight heparin

**Table S5a:** Peak values of aFXa by initial dose of low-molecular-weight heparin

|  | Low dose (n = 30)^a^ | Intermediate dose (n = 95)^b^ | High dose  (n = 22)^c^ | *p* value^d^ |
| --- | --- | --- | --- | --- |
| Minimum aFXa (kIU/L) | 0.31 (0.27 to 0.48) | 0.41 (0.30 to 0.55) | 0.46 (0.38 to 0.58) | 0.10 |
| Median aFXa (kIU/L) | (n = 21)  0.42 (0.31 to 0.53) | (n = 86)  0.51 (0.35 to 0.64) | (n = 17)  0.58 (0.40 to 0.75)^e^ | 0.06 |
| Maximum aFXa (kIU/L) | 0.50 (0.30 to 0.66) | 0.57 (0.42 to 0.78) | 0.51 (0.40 to 0.75)^e^ | 0.16 |

^a^ Tinzaparin, 2500–4500 IU daily, dalteparin, 2500–5000 IU daily, or enoxaparin, ≤ 40 mg daily

^b^ Tinzaparin, > 4500 IU daily to < 175 IU/kg of body weight daily, or dalteparin, > 5000 IU daily to < 200 IU/kg of body weight daily, or enoxaparin, > 40 mg but < 1 mg/kg of body weight daily.
^c^ Tinzaparin, ≥ 175 IU/kg of body weight per daily, dalteparin, ≥ 200 IU/kg of body weight daily, or enoxaparin, ≥ 1 mg/kg of body weight daily.

^d^ *p* values for differences across groups were obtained using Kruskal-Wallis test

^e^ Only 17 of 22 patients contributed to the median, that is, not the same population that contributed to the minimum and maximum values. Therefore, it is possible for the median to be higher than the maximum value.

Six patients in the peak group had no initial thromboprophylaxis or missing initial doses

**Table S5b:** Trough values of aFXa by initial dose of low-molecular-weight heparin

|  | Low dose (n= 22)^a^ | Intermediate dose (n = 194)^a^ | High dose  (n = 71)^c^ | *p* value^d^ |
| --- | --- | --- | --- | --- |
| Minimum aFXa (kIU/L) | 0.18 (0.13 to 0.30) | 0.21 (0.13 to 0.30) | 0.29 (0.17 to 0.48) | 0.005^e^ |
| Median aFXa (kIU/L) | (n = 13)  0.23 (0.08 to 0.42) | (n = 176)  0.29 (0.20 to 0.41) | (n = 66)  0.43 (0.28 to 0.57) | <0.001^f^ |
| Maximum aFXa (kIU/L) | 0.41 (0.26 to 0.70) | 0.33 (0.22 to 0.54) | 0.51 (0.38 to 0.62) | 0.002^g^ |

^a^ Tinzaparin, 2500–4500 IU daily, dalteparin, 2500–5000 IU daily, or enoxaparin, ≤ 40 mg daily^b^ Tinzaparin, > 4500 IU daily to < 175 IU/kg of body weight daily, or dalteparin, > 5000 IU daily to < 200 IU/kg of body weight daily, or enoxaparin, > 40 mg but < 1 mg/kg of body weight daily.
^c^ Tinzaparin, ≥ 175 IU/kg of body weight per daily, dalteparin, ≥ 200 IU/kg of body weight daily, or enoxaparin, ≥ 1 mg/kg of body weight daily.

^d^ *p* values for differences across groups were obtained using Kruskal-Wallis test and Dunns test adjusted by the Bonferroni method

^e^ Significant differences between intermediate and high dose

^f^ Significant differences between low and high dose and intermediate and high dose

^g^ Significant difference between intermediate and high dose

Thirteen patients in the trough group had no initial thromboprophylaxis or missing initial doses.

## **Fig. S1.** Distribution of patients’ minimum and maximum anti-Factor Xa values during intensive care stay with proportions of outcomes of thromboembolism, bleeding, major bleeding, and death.

^^

**Fig. S1.** Distribution of anti-Factor Xa peak and trough values and the outcomes of thromboembolism, bleeding, major bleeding, and death in the 408 patients with critical COVID-19 admitted to the intensive care unit from March 2020 to May 2021. Minimum values are visualized against the event of thromboembolism, median values against the event of death, and maximum values against the events of bleeding, major bleeding and death. Green indicates no event, blue indicates thromboembolism, pink indicates bleeding, orange indicates major bleeding, and black indicates death.

## **Fig. S2.** Scatterplot of the correlation between anti-Factor Xa values and estimated glomerular filtration rate.

**Fig. S2.** The correlation between anti-Factor Xa values and estimated glomerular filtration rate (eGFR) in 406 patients with critical COVID-19 admitted to the intensive care unit from March 2020 to May 2021. For the minimum, median and maximum peak value, nonsignificant correlations of -0.1, -0.13, and -0.03 were observed (*p* = 0.21, 0.13 and 0.68). Additionally, for the minimum trough value, a nonsignificant correlation of -0.10 was observed (*p* = 0.09). Median and maximum trough values had a significant correlation with a decrease in eGFR increasing trough values *(p* = 0.009 and 0.0008). However, the correlations of -0.2 for median values and -0.23 for maximum values indicated a modest effect of eGFR on trough values.

## **Fig. S3.** Anti-Factor Xa peak values and association with outcomes adjusted for estimated glomerular filtration rate.

**Fig. S3.** The association between anti-Factor Xa peak values and death, thromboembolism, bleeding and major bleeding with adjustment for baseline estimated glomerular filtration rate (eGFR). The black line indicates the risk for patients with an eGFR of 60 ml/min/1.73 m^2^, the orange line indicates the risk for patients with an eGFR of 90 ml/min/1.73 m^2^, the blue line indicates the risk for patients with an eGFR of 120 ml/min/1.73 m^2^, and the shaded area indicates 95% confidence intervals. The figures illustrate peak values when summarized as minimum values during intensive care (153 peak values), median values during the first 14 days of intensive care (126 peak values), and maximum values during intensive care (153 peak values).

The minimum, median and maximum values were not associated with death (*p* = 0.30, 0.84 and 0.43). Lower minimum, median and maximum values were associated with a higher risk of thromboembolism (*p* = 0.01, 0.01 and 0.001). The minimum, median and maximum values were not associated with bleeding (*p* = 0.81, 0.40 and 0.37) or major bleeding (*p* = 0.30, 0.51 and 0.21).

## **Fig. S4.** Anti-Factor Xa trough values and association with outcome adjusted for estimated glomerular filtration rate.

**Fig. S4.** The association between anti-Factor Xa trough values and death, thromboembolism, bleeding and major bleeding with adjustment for baseline estimated glomerular filtration rate (eGFR). The black line indicates the risk for patients with an eGFR of 60 ml/min/1.73 m^2^, the orange line indicates the risk for patients with an eGFR of 90 ml/min/1.73 m^2^, the blue line indicates the risk for patients with an eGFR of 120 ml/min/1.73 m^2^, and the shaded area indicates 95% confidence intervals. The figures illustrate trough values when summarized as minimum values during intensive care (300 trough values), median values during the first 14 days of intensive care (266 trough values), and maximum values during intensive care (300 trough values).

Not the minimum, but higher median and maximum values were associated with an increased risk of death (*p* = 0.10, 0.05 and 0.01). The minimum, median and maximum values were not associated with thromboembolism (*p* = 0.34, 0.42 and 0.14). Not the minimum or median, but a higher maximum value was associated with an increased risk of bleeding (*p* = 0.14, 0.73 and 0.04), but no significant associations were found between trough values and major bleeding (*p* = 0.77, 0.56 and 0.06).

## **Fig. S5.** Distribution of anti-Factor Xa values by type of low-molecular-weight heparin.

**Fig. S5.** The distribution of anti-Factor Xa values by patients treated with different types of low-molecular-weight heparins among the 408 patients with critical COVID-19 admitted to the intensive care unit from March 2020 to May 2021. Pink indicates treatment with dalteparin, green indicates treatment with enoxaparin, and blue indicates treatment with tinzaparin.
